# Supplementary material for: Nectar non-protein amino acids (NPAAs) do not change nectar palatability but enhance learning and memory in honey bees
Source: Sci Rep. 2021 Jun 3;11:11721. doi: 10.1038/s41598-021-90895-z (PMC8175726; doi:10.1038/s41598-021-90895-z)
Supplement: Supplementary file 1 — Supplementary Information. [file 41598_2021_90895_MOESM1_ESM.pdf]

SUPPLEMENTARY INFORMATION

Nectar non-protein amino acids (NPAAs) do not change nectar palatability but enhance learning and memory in honey bees

Daniele Carlesso[1], Stefania Smargiassi[2], Elisa Pasquini[3], Giacomo Bertelli[3], David Baracchi[3]

[1] *Department of Biological Sciences, Macquarie University, Sydney, NSW 2109, Australia*

[2] *Department of Life Sciences and Systems Biology, University of Turin, Via Accademia Albertina 13, Turin 10123, Italy*

[3] *Department of Biology, University of Florence, Via Madonna del Piano, 6, 50019 Sesto Fiorentino, Italy*

Results

Exp 3 – Influence of NPAAs on feeding and mortality

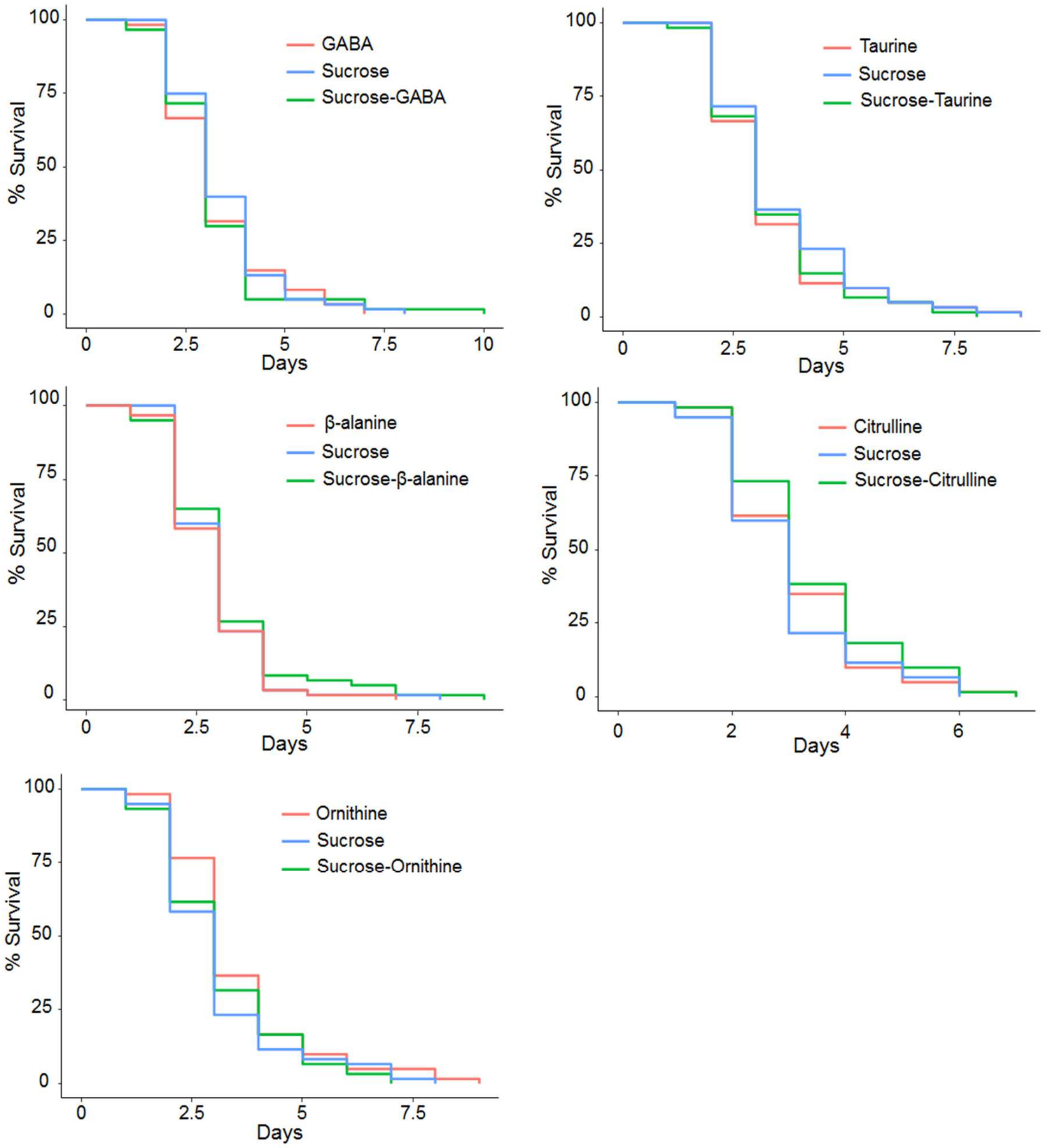

Figure S1: NPAAs did not affect caged honey bee survival. *Cumulative survival of bees kept in caged conditions under three different feeding regimes for a period of 10 days: Sucrose only (S-S); NPAA-laced solution only (NPAA-NPAA); Sucrose and NPAA-laced solution (S-NPAA). None of the NPAAs was a significant predictor of mortality in any feeding regime (Log-rank Mantel Cox test, GABA: p=0.68; β-ALA: p=0.52; TAU: p=0.68; CIT: p=0.20; ORN: p=0.25).*

Exp 4 – Contextual absolute olfactory learning

For all *unpaired* groups, we observed no significant increase in responses during conditioning apart from β-alanine (GLMM, *trial*: GABA, χ^2=0.15, df=1, p=0.70; β-ALA, χ^2=4.32, df=1, p=0.04; TAU, χ^2=1.26, df=1, p=0.26; CIT, χ^2=0.19, df=1, p=0.66; ORN, χ^2=0.59, df=1, p=0.44, Fig. 4), nor an effect of the treatment (GLMM, *treat:* GABA, χ^2=0.04, df=1, p=0.84; β-ALA, χ^2=0.13, df=1, p=0.71; TAU, χ^2=0.01, df=1, p=0.93; CIT, χ^2=0.0, df=1, p=0.99; ORN, χ^2=0.001, df=1, p=0.98, Fig. 4 in the main document). Accordingly, bees in the *unpaired* groups did not differ in acquisition scores for any of the NPAAs (Mann-Whitney U test, *ACQS:* GABA, W=771, p=0.92; β-ALA, W=818, p=0.39; TAU, W=740, p=0.15; CIT, W=861, p=0.34; ORN, W=722, p=0.1). These results thus confirmed the occurrence of a true associative learning phenomenon in the *paired* groups.

In all the *unpaired* groups NPAAs did not alter bees' responses to the conditioned odorant (χ^2 test, *CS:* GABA, χ^2=0.14, p=0.71; β-ALA, χ^2=0.37, p=0.54; TAU, χ^2=0.38, p=0.55; CIT, χ^2=2.67, p=0.10; ORN, χ^2=0.24, p=0.62, Fig. 4) nor to the novel odorant (χ^2 test, *NOd:* GABA, χ^2=0.90, p=0.34; β-ALA, χ^2=0.99, p=0.32; TAU, χ^2=0.0003, p=0.99; CIT, χ^2=1.95, p=0.16; ORN, χ^2=2.00, p=0.16, Fig. 4). Accordingly, we found no difference in the proportion of experimental and control bees showing CS-specific memory for any of the NPAAs (χ^2 test, *specific memory*: GABA, χ^2=0.14, p=0.71; β-ALA, χ^2=0.37, p=0.54; TAU, χ^2=1.04, p=0.31; CIT, χ^2=1.77, p=0.18; ORN, χ^2=0.001, p=0.98, Fig. 4 in the main document).

Exp 5 – Post-feeding absolute olfactory learning

Bees belonging to the *unpaired* groups did not increase their responses over training except with β-alanine (GLMM, *trial:* GABA, χ^2=3.56, df=1, p=0.06; β-ALA, χ^2=4.87, df=1, p=0.03; TAU, χ^2=0.38, df=1, p=0.54; CIT, χ^2=1.26, df=1, p=0.26; ORN, χ^2=2.87, df=1, p=0.1, Fig. 5 in the main document). However, in all the *unpaired groups,* including β-alanine, pre-feeding did not alter the responses to the CS (GLMM, *treat:* GABA: χ^2=0.04, df=1, p=0.84; β-ALA: χ^2=1.88, df=1, p=0.17; TAU*:* χ^2=0.0004, df=1, p=0.98; CIT: χ^2=0.06, df=1, p=0.81; ORN: χ^2=0.053, df=1, p=0.82, Fig. 5 in the main document). Accordingly, experimental and control pre-fed bees did not differ in their ACQS (Mann-Whitney U test, *ACQS*: GABA: W=529, p=0.69; β-ALA: W=559, p=0.15; TAU: W=666, p=1; CIT: W=841, p=0.98; ORN: W=665, p=0.68). Overall, the results confirmed that true associative learning occurred in the *paired* groups.

In the *unpaired* groups, no NPAA altered the responses to the CS (χ^2 test, GABA: χ^2=0.94, p=0.16; β-ALA: χ^2=0.53, p=0.47; TAU: χ^2=0.001, p=0.97; CIT: χ^2=1.61, p=0.20; ORN: χ^2=0.56, p=0.45) or to the NOd (χ^2 test, GABA: χ^2=0.72, p=0.40; β-ALA: χ^2=1.33, p=0.25; TAU: χ^2=0.001, p=0.98; CIT: χ^2=1.77, p=0.18; ORN: χ^2=0.11, p=0.74, Fig. 5). Accordingly, *unpaired* experimental and control bees did not differ in CS-specific memory for any of the NPAAs (χ^2 test, GABA: χ^2=0.24, p=0.63; β-ALA: χ^2=0.049, p=0.83; TAU: χ^2=0.38, p=0.54; CIT: χ^2=0.45, p=0.50; ORN: χ^2=0.21, p=0.64, Fig. 5 in the main document).
